# Effects of Posterior Spinal Correction and Fusion on Postural Stability in Patients with Adolescent Idiopathic Scoliosis

**DOI:** 10.3390/jcm12010270

**Published:** 2022-12-29

**Authors:** Satoshi Osuka, Hideki Sudo, Katsuhisa Yamada, Hiroyuki Tachi, Kentaro Watanabe, Fuma Sentoku, Takeshi Chiba, Norimasa Iwasaki, Masahiko Mukaino, Harukazu Tohyama

**Affiliations:** 1 Faculty of Health Sciences, Hokkaido University, Sapporo 060-0812, Hokkaido, Japan; 2 Department of Rehabilitation, Hokkaido University Hospital, Sapporo 060-8648, Hokkaido, Japan; 3 Department of Advanced Medicine for Spine and Spinal Cord Disorders, Faculty of Medicine and Graduate School of Medicine, Hokkaido University, Sapporo 060-8638, Hokkaido, Japan; 4 Department of Orthopaedic Surgery, Hokkaido University Hospital, Sapporo 060-8638, Hokkaido, Japan; 5 Department of Orthopaedic Surgery, Eniwa Hospital, Eniwa 061-1449, Hokkaido, Japan; 6 Department of Rehabilitation Medicine, Hokkaido University Hospital, Sapporo 060-8648, Hokkaido, Japan

**Keywords:** adolescent idiopathic scoliosis, anatomical spinal correction, postural stability, center of pressure, force plate

## Abstract

The present study aimed to assess the effects of posterior spinal correction and fusion on postural stability in patients with adolescent idiopathic scoliosis (AIS). The study included 41 female patients with AIS at our institution. All patients performed three 10 s single-leg standing trials on a force plate. The center of pressure (COP) was measured preoperatively, and at 1 week and 6 months postoperatively. The postural stability parameters were absolute minimum time-to-boundary (TTB), mean of the minimum TTB, mean COP velocity, standard deviation, range, and 95% confidence ellipse area. One-way repeated analysis of variance or Friedman test was applied to the postural stability parameters. Multiple comparisons were performed using the Bonferroni correction. The absolute minimum TTB and the mean minimum TTB showed a significant increase 6 months post-operation as compared to preoperatively and 1 week postoperatively. The COP velocity significantly decreased at 6 months post-operation compared to preoperatively and 1 week postoperatively. These changes in postural stability indicate that spinal correction and fusion can be considered to improve postural stability during single-leg standing tests in the postoperative period.

## 1. Introduction

Adolescent idiopathic scoliosis (AIS) is one of the most common spinal deformities and is characterized by lateral spinal curvature with axial rotation and, typically, hypokyphosis of the thoracic spine [1]. AIS patients show different postural control and patterns [2,3,4,5]. The change in posture is a possible reason for abnormal load upon the spine and secondary deformity. Patients with AIS with a Cobb angle greater than 40 degrees may require surgical treatment and spinal deformity correction, leading to changes in postural balance.

To treat various types of AIS, the posterior spinal correction and fusion using segmental pedicle screw instrumentation is often performed. Preserving and restoring a normal sagittal alignment is crucial for anatomical spinal correction, because patients with thoracic AIS are frequently hypokyphotic [6,7]. Sudo et al. developed a four-dimensional (4D) anatomical spinal reconstruction technique that involves the use of spatiotemporal deformity prediction to preoperatively calculate the postoperative apex of thoracic kyphosis in order to achieve an anatomically correct spinal curvature [8,9,10,11]. In the surgical technique, two rods are identically bent according to the desired postoperative anatomical spine, with the thoracic apex anticipated to be located at T6-T8, when viewing standing sagittal radiographs [8,9,11]. This technique could improve the Scoliosis Research Society questionnaire score and radiographic data of the Cobb angle, thoracic hypokyphosis, and location of the thoracic kyphosis apex [8,9,11]. However, the effects of this surgical technique on postural stability in patients with AIS are still unknown.

Time-to-boundary (TTB) is a recently developed multidimensional measure to evaluate postural stability that incorporates body sway with the base of support [12,13,14]. This outcome records the position and speed of the center of pressure (COP) in relation to the base of support, indicating how much time remains before the COP comes into contact with the base of support boundary. Therefore, TTB can highlight the crucial components of postural control and record the most difficult period during the stance period. Hertel et al. [13] suggested that TTB measures capture a component of postural stability that is distinct from conventional COP-based measures because there was a weak correlation between TTB results and conventional COP-based results. Additionally, it has been shown that TTB is more effective at identifying individuals with chronic ankle instability than conventional COP-based assessments [14]. Compared to conventional COP-based measures, TTB offers a deeper understanding of the spatiotemporal features of postural control since it takes into consideration the direction and location of the COP relative to the foot border [14].

The purpose of this study was to evaluate the postural stability of patients with AIS who underwent surgical treatment with the 4D anatomical spinal reconstruction technique, and to determine the effect of surgical treatment using a longitudinal study with TTB measures. Quantifying differences in postural stability between pre- and post-surgery with the 4D anatomical spinal reconstruction technique for participants with AIS may assist in evaluating the effectiveness of this surgery.

## 2. Materials and Methods

### 2.1. Subjects

**Figure 1:**
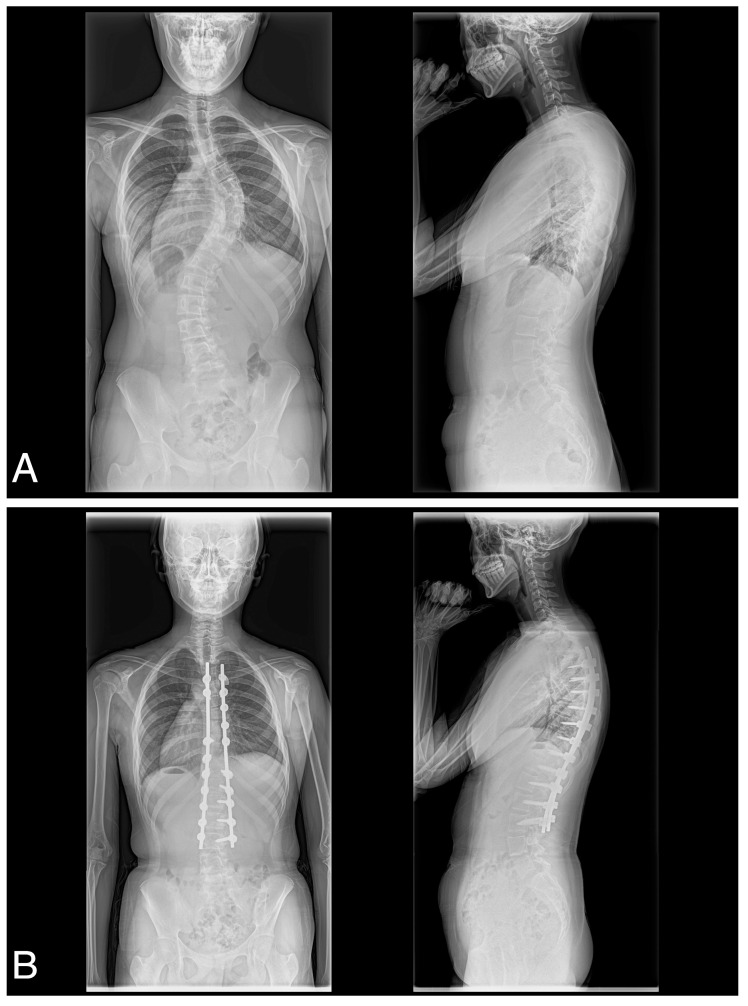
(**A**) Preoperative posteroanterior and lateral and (**B**) postoperative posteroanterior and lateral radiographs obtained of a 17-year-old female with Lenke type 1C scoliosis.

A consecutive series of 41 female patients with AIS with Lenke type 1A (*n* = 18), 1B (*n* = 7), 1C (*n* = 7), 2A (*n* = 3), 3C (*n* = 4), 4C (*n* = 1), or 6C (*n* = 1) participated in the present study (Table 1). All patients underwent posterior spinal correction and fusion using the 4D anatomical spinal reconstruction technique to correct AIS (Figure 1) [8]. Patients with syndromic, neuromuscular, or congenital scoliosis were excluded. In addition, because the 4D anatomical spinal reconstruction technique was developed to create postoperative anatomical thoracic kyphosis, we have excluded Lenke 5C main thoracolumbar/lumbar AIS curves. All patients were included in the follow up of the study. All patients in this study gave written, informed consent before participating, and the Institutional Review Board of the authors’ associated institutions authorized the study.

### 2.2. Surgical Technique

The 4D anatomical spinal reconstruction technique was previously described in detail [8]. Briefly, facetectomies are performed on all levels except for the lowest intervertebral segment. Two rods are identically bent based on the desired postoperative anatomical thoracic kyphosis, where the apex is anticipated to be located at T6–T8. Two different spinal rod shapes or categories have been defined to cover all presenting anatomies. The single curve rod is used when the lowest instrumented vertebra (LIV) is L1 or above. The apex is located at T6–T8 level, while the thoracolumbar region remains straight. If the LIV is L2 or L3, the shape is of the double curve type. Similar to the single curve, the cranial apex is created. Eleven shapes of pre-bent, notch-free, cobalt chrome alloy rods are available in Japan. Once the two spinal rods are connected to all polyaxial screw heads, the rods are simultaneously rotated.

Most patients were hospitalized for approximately 10 days, and during their hospital stay, they were trained in basic activities of daily living such as getting up, walking, and ascending and descending stairs, according to subjective pain intensity. After being discharged, no medically supervised rehabilitation was offered.

### 2.3. Postural Stability Parameters

A force plate (MG-1060; Anima Inc., Tokyo, Japan) was used to calculate the COP coordinates in the anteroposterior and mediolateral directions during single-leg standing test. With eyes open, the patients attempted to stand quietly on a single leg for 10 s with their bare foot on the right side on the force plate. The test was performed three times: preoperative, at 1 week postoperatively, and at 6 months postoperatively. The stance foot was placed at the same position on the force plate in all trials. A total of 1000 COP data points were calculated for each trial’s force plate data, which were recorded at a sampling rate of 100 Hz. A fourth-order low-zero-lag low-pass filter with a cutoff frequency of 5 Hz was then used to filter the COP data.

**Figure 2:**
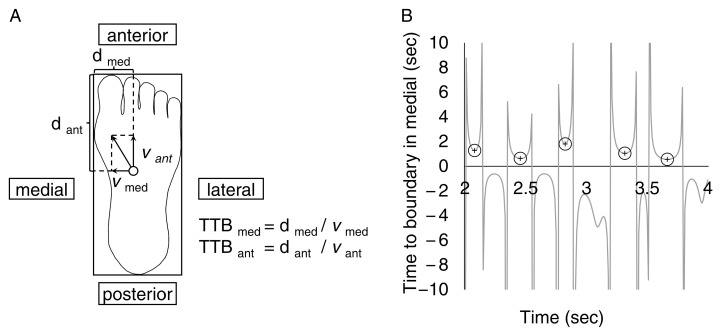
(**A**) The time-to-boundary (TTB) in each direction equals the distance between the center of pressure (open circle) and the impending boundary of the foot divided by the corresponding center of pressure velocity. Suppose the COP was moving in the anterior and medial directions. In that case, the TTB is calculated by dividing the distance to the medial and anterior boundaries of the foot by the velocity. (**B**) A typical center of pressure displacement data during single-leg standing tests. The time-to-boundary minima in medial (represented by circles) are identified at the valleys.

TTB measurements were made with reference to previous reports [13,15]. To separate the medial, lateral, anterior, and posterior components of the COP, the foot was modeled as a rectangle based on where it was on the force plate. For the TTB in the medial direction, the COP for the mediolateral position and velocity was used. The distance between the COP and the medial foot border was recorded. The distance was divided by the velocity to the medial border of the foot to calculate the time taken to reach the medial border of the foot when the COP continued to move in the same direction without changing its velocity (Figure 2A). A time series of TTB in the medial, lateral, anterior, and posterior directions was generated. In each trial, we gathered TTB measurements in the valleys by using the “isocalmin” function in MATLAB 2022a to identify appropriate points (Figure 2B). The minima that were larger than the mean plus 2SD were eliminated [16]. The absolute minimum and mean of the minimum in the medial, lateral, anterior, and posterior directions were calculated. Although these two measurements represent the temporal margin to the boundary of support, there is no significant clinical difference between these measurements. In addition, the other COP measures were the mean velocity of the COP excursions, the standard deviation of the COP excursions, the range of the COP excursions in the mediolateral and anteroposterior directions, and the area of the 95% confidence ellipse of the COP movement. Three trials of the single-leg standing test were performed in one measurement, and the average of the obtained values was used for later analysis.

### 2.4. Radiographic Parameters

Standing long-cassette radiographs in sagittal and coronal plane of all patients were assessed for several parameters prior to surgery, as well as at 1 week and 6 months after surgery. All radiographic data were analyzed by two board-certificated spine surgeons. Our internal studies of inter/intrarater reliability have shown excellent κ statistics for all continuous measures (0.97–0.99). The proximal thoracic (PT), main thoracic (MT), and thoracolumbar/lumbar (TL/L) curves’ Cobb measurements were taken in the coronal planes. Sagittal plane data, thoracic kyphosis (T5–T12), and lumbar lordosis (L1-S1) were also measured [11].

The C7 coronal plumb line’s lateral deviation from the center sacral vertical line (CSVL) served as a proxy for global coronal balance. Sagittal balance was measured by the absolute distance between the C7 plumb line and the S1 posterior superior corner, which was then represented as the sagittal vertical axis (SVA). The MT apical vertebral translation was calculated for regional alignment as the separation between the C7 plumb line and the geometric center of the apical vertebrae [11]. For the TL/L apical vertebral translation, the distance between the geometric center of the apical vertebrae and the CSVL was calculated [11].

### 2.5. Statistical Analysis

IBM SPSS Statistics 28 (IBM, Chicago, IL, USA) was used to conduct the statistical analysis. The Shapiro–Wilk test was used to examine normality. One-way repeated analysis of variance or Friedman test was used to examine the differences between preoperative, 1 week postoperative, and 6 months postoperative periods. Post hoc analysis was performed using the Bonferroni correction for pairwise comparisons. The statistical significance level was set at *p* = 0.05.

## 3. Results

### 3.1. Postural Stability Parameters

**Figure 3:**
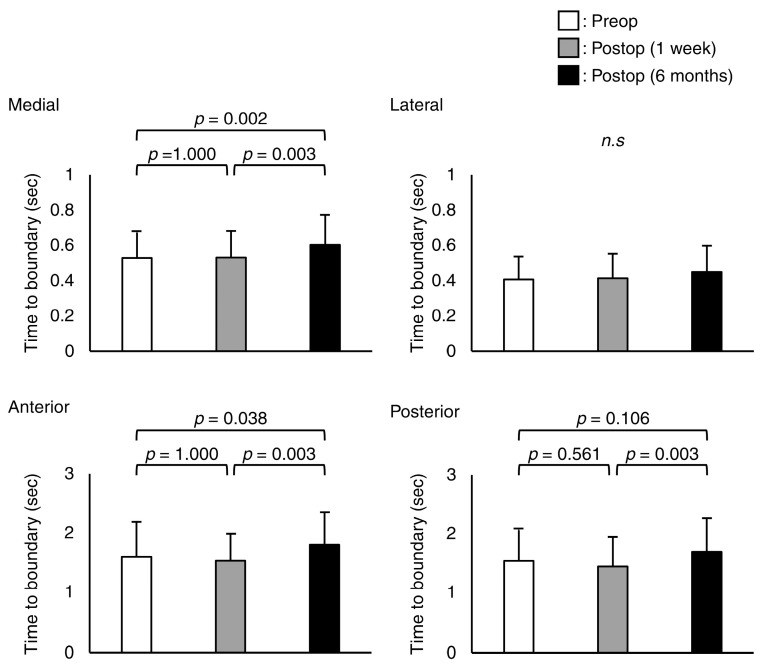
Mean and standard deviation values of the absolute minimum of the time-to-boundary in medial, lateral, anterior, and posterior directions. *n.s* means not significant.

In the medial, anterior, and posterior directions, the absolute minimum TTB measurements were significantly different (*p* < 0.001, *p* = 0.049, and *p* = 0.003, respectively) (Figure 3). In the post hoc test, the absolute minimum values of TTB for the medial and anterior at 6 months postoperatively were higher than those in the preoperative and 1 week postoperative trials (*p* = 0.002, *p* = 0.003, *p* = 0.038, and *p* = 0.003, respectively). The absolute minimum values of posterior TTB at 6 months postoperatively were higher than that at 1 week postoperatively (*p* = 0.003).

**Figure 4:**
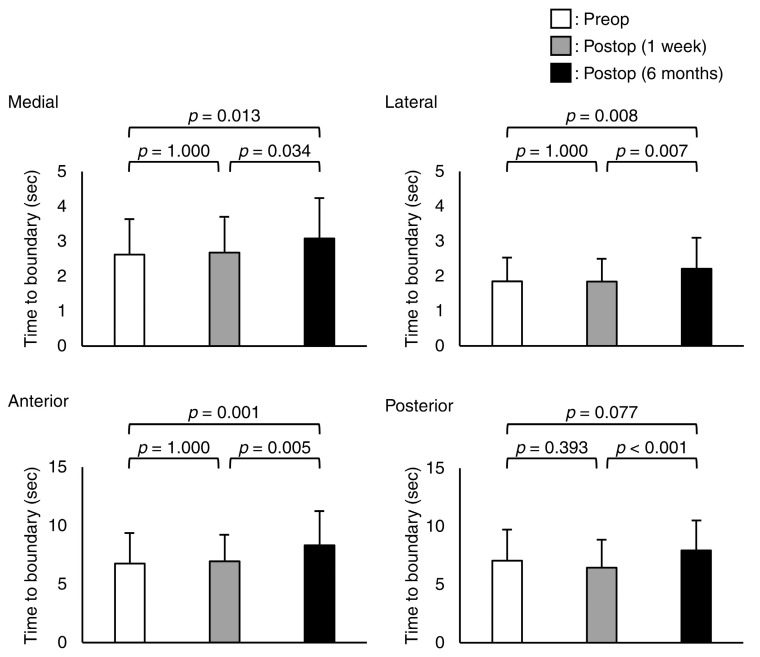
Mean and standard deviation values of the mean minimum of the time-to-boundary in medial, lateral, anterior, and posterior directions.

There were significant differences in the mean minimum TTB in the medial, lateral, anterior, and posterior directions (*p* = 0.004, *p* = 0.043, *p* = 0.005, and *p* = 0.001, respectively) (Figure 4). The mean minimum TTB for the medial, lateral, and anterior directions at 6 months post-operation was higher than the preoperative and 1 week post-surgery values (*p* = 0.013, *p* = 0.034, *p* = 0.008, *p* = 0.007, *p* = 0.001, and *p* = 0.005, respectively). The mean minimum posterior TTB at 6 months post-operation was higher than that at 1 week post-operation trials (*p* < 0.001).

For the conventional COP measures, there were significant differences in COP velocities for the mediolateral and posteroanterior directions (*p* < 0.001 and *p* < 0.001, respectively) (Table 2). The post hoc analysis revealed that the COP velocity in the mediolateral and posteroanterior direction at 6 months postoperatively was higher than preoperatively and at 1 week postoperatively (*p* < 0.001, *p* < 0.001, *p* < 0.001, and *p* < 0.001, respectively). There was a significant difference in standard deviation for the mediolateral direction (*p* = 0.015). In the post hoc test, the standard deviation for the mediolateral direction at 6 months postoperatively was lower than that at 1 week postoperatively (*p* = 0.017).

### 3.2. Radiographic Parameters

The radiographic parameters of coronal plane data, sagittal plane data, balance parameters, and translational data have been summarized in Table 3. There were significant differences in the PT, MT, and TL/L curves (*p* < 0.001, *p* < 0.001, and *p* < 0.001, respectively). At 1 week and 6 months postoperatively, the mean PT, MT, and TL/L curves were significantly higher than the preoperative ones (*p* < 0.001, *p* < 0.001, *p* < 0.001, *p* < 0.001, *p* < 0.001, and *p* < 0.001, respectively). The mean MT curve correction rate was 79.5% at 1 week postoperatively. At 6 months postoperatively, the correction rate and correction angle loss for the MT curve were 77.8% and 1.04°, respectively. The mean PT curve correction rate was 58.3% at 1 week postoperatively. At 6 months postoperatively, the correction rate and the correction angle loss for the PT curve were 60.3% and 0.36°, respectively. The mean TL/L curve correction rate was 76.2% at 1 week postoperatively. At 6 months postoperatively, the correction rate and correction angle loss for the TL/L curve were 73.0% and 0.68°, respectively.

In the sagittal plane data, there were significant differences in the thoracic kyphosis and lumbar lordosis (*p* < 0.001 and *p* < 0.001, respectively). The 1 week and 6 months mean thoracic kyphosis was significantly higher than the preoperative mean (*p* < 0.001 and *p* < 0.001, respectively). The 6 months mean lumbar lordosis was significantly higher than the preoperative and 1 week postoperative mean (*p* = 0.029 and *p* < 0.001, respectively).

There were significant differences in C7 translation from CSVL, SVA, and thoracic apical vertebral translation (*p* = 0.005, *p* = 0.018, and *p* < 0.001, respectively). The C7 translation from CSVL was significantly decreased at 6 months postoperatively compared to preoperatively and at 1 week postoperatively (*p* < 0.001 and *p* = 0.017, respectively). The SVA was significantly decreased at 1 week postoperatively compared to preoperatively (*p* = 0.021). The thoracic apical vertebral translation decreased significantly at 1 week and 6 months postoperatively compared to that preoperatively (*p* < 0.001 and *p* < 0.001, respectively). There were no significant differences in the TL/L apical vertebral translation (*p* = 0.779).

## 4. Discussion

In this study, we investigated the postural stability of AIS patients who underwent surgical operation with the 4D anatomical spinal reconstruction technique from preoperative to 6 months postoperative. This study revealed that postural stability was better 6 months postoperatively than preoperatively and 1 week postoperatively.

Patients with AIS who underwent surgery with the 4D anatomical spinal reconstruction technique had significantly higher values for TTB measures in the medial, lateral, anterior, and posterior directions 6 months post-operation. Decrease in TTB measurements suggest deterioration of the postural control mechanism. The results of this study indicate that while patients with AIS preoperatively and at 1 week postoperatively were controlling their balance, they controlled it in a way that brought the COP closer to the domain’s medial, lateral, anterior, and posterior stability limits compared to 6 months later. Similar to the TTB parameters, a significant decrease in COP velocity was observed 6 months postoperatively, suggesting that the change in TTB parameters was due to the change in COP velocity.

Three-dimensional spinal deformities due to AIS have been reported to result in asymmetric trunk muscle activity [17,18] and impairments in proprioceptive disorders [19]. These changes in body function are presumed to impair postural stability [2,3,4,5]. Although these changes in postural stability are thought to be affected by spinal correction and fusion, previous studies investigating the impact of surgical treatment for AIS on postural stability have not reached a consensus. Valles et al. [20] demonstrated that patients with AIS had significantly better postural stability 1 year postoperatively than preoperatively. However, de Abreu et al. [5], Schimmel et al. [21], O’beirne et al. [22], and St-Georges et al. [23] demonstrated that improved spinal morphology has no impact on postural stability in patients with AIS. Despite the above results, we found a change in the postural stability of patients with AIS 6 months postoperatively compared to preoperatively. A direct comparison between studies is rather difficult because a variety of study methodologies have been used with a myriad of outcome measures. However, the results of this study would be explained by the characteristics of the surgical techniques used.

The objective of surgical management of AIS is to maximize deformity correction and achieve coronal and sagittal balance [24,25]. Typical patients with thoracic AIS exhibit thoracic hypokyphosis in addition to coronal deformity [6], and further lordosis has been reported to occur after corrective surgery [26,27,28]. In contrast, Sudo et al. [11] reported that the 4D anatomical spinal reconstruction technique can improve hypokyphosis and the location of thoracic apex in patients with thoracic AIS. These results indicate that spinal correction and fusion using the 4D anatomical spinal reconstruction technique could be used to obtain anatomical spinal reconstruction in thoracic AIS. In this study, the results of radiographic parameters also showed that the thoracic kyphosis angles (T5–T12) were significantly increased at 6 months postoperatively compared to preoperatively. In addition, the surgical correction rates in this study were greater than those of the previous study [23], in which there were no significant differences in postural stability between preoperative and postoperative 6 months. These results suggest that spinal correction and fusion using the 4D anatomical spinal reconstruction technique may have improved postural stability at 6 months postoperatively through anatomical spinal correction rather than a natural result that postural stability improves over time. The improvement of TTB can also be due to postoperative rehabilitation or the fact that the spine is fused and does not allow the same amount of correctional movement in the body with a lower trajectory of COP. Patients with neurological diseases sometimes show very small trajectories of COP as well as postural sway because they do not dare to move close to their boundaries. Clinical interpretation of TTB should take these possibilities into account.

This study had some limitations. First, the follow-up period was limited to 6 months. Therefore, long-term changes in postural stability in patients who underwent posterior spinal correction and fusion using the 4D anatomical spinal reconstruction technique are unknown. Adding the value of at least two years after surgery can improve the completion of this study. Second, there was a lack of follow-up on their daily activities and activity levels. Although all patients were prohibited from sport activities during the relevant period, it is unknown what kind of daily activities they engaged in after surgery. Third, there is no electromyographic evaluation that analyzed muscle activation patterns before and after surgery. There is a possibility that changes in postural stability parameters were a result of changes in trunk muscle activity, and analysis of muscle activity may provide a more detailed explanation of the changes in postural stability observed in this study. Last, in this study, all patients underwent the 4D anatomical spinal reconstruction technique to obtain thoracic kyphosis. Based on the ethical reasons, it is impossible to compare the postoperative postural stability between patients who gain the thoracic kyphosis and those who did not improve thoracic kyphosis using different surgical techniques.

## 5. Conclusions

The patients with AIS who had posterior spinal correction and fusion using the 4D anatomical spinal reconstruction technique had a significantly longer TTB of their COP at 6 months following surgery. These changes in postural stability indicate that spinal correction and fusion using this technique may improve postural stability during single-leg standing tests in the postoperative period.

## Figures and Tables

**Table 1:** Patient demographic data.

	Mean (Standard Deviation)	Range
Age at surgery, yrs	14.7 (2.0)	11–18
Height at surgery, cm	157.1 (6.6)	140.0–171.0
Weight at surgery, kg	47.2 (7.8)	29.5–62.5
Risser sign	3.7 (1.5)	0–5
Cobb length (upper end–lower end vertebra)	7.6 (0.9)	6–10
Instrumentation length (segments)	10.9 (1.6)	6–13
Operation time, min	254.3 (52.4)	126–402

The values are given as the mean (standard deviation).

**Table 2:** Mean and standard deviation value for the center of pressure parameters preoperatively and postoperatively.

	Preop	Postop (1 Week)	Postop (6 Months)	Overall *p*	Post Hoc Test *p*		
					Preop to Postop (1 Week)	Preop to Postop (6 Months)	Postop (1 Week) to Postop (6 Months)
Velocity, cm/s							
Mediolateral	2.34 (0.63)	2.28 (0.58)	2.04 (0.51)	<0.001	1.000	<0.001	<0.001
Anteroposterior	2.04 (0.63)	2.07 (0.55)	1.77 (0.53)	<0.001	1.000	<0.001	<0.001
Standard deviation, cm							
Mediolateral	0.44 (0.08)	0.47 (0.09)	0.44 (0.08)	0.015	0.102	1.000	0.017
Anteroposterior	0.61 (0.15)	0.68 (0.25)	0.63 (0.17)	0.249	-	-	-
Range, cm							
Mediolateral	2.07 (0.34)	2.13 (0.37)	2.02 (0.38)	0.303	-	-	-
Anteroposterior	2.74 (0.63)	3.00 (0.93)	2.79 (0.67)	0.303	-	-	-
95% confidence ellipse area, cm^2^	4.98 (1.94)	5.95 (3.24)	5.10 (2.02)	0.109	-	-	-

The values are given as the mean (standard deviation).

**Table 3:** Mean and standard deviation values for the radiographic parameters preoperatively and postoperatively.

	Preop	Postop (1 Week)	Postop (6 Months)	Overall *p*	Post Hoc Test *p*		
					Preop to Postop (1 Week)	Preop to Postop (6 Months)	Postop (1 Week) to Postop (6 Months)
Coronal plane data, degree							
Proximal thoracic curve	26.9 (8.5)	11.4 (6.3)	11.1 (6.8)	<0.001	<0.001	<0.001	1.000
Main thoracic curve	53.5 (9.1)	11.6 (7.3)	12.6 (6.4)	<0.001	<0.001	<0.001	1.000
Thoracolumbar/lumbar curve	36.1 (12.6)	9.1 (6.0)	9.8 (6.2)	<0.001	<0.001	<0.001	1.000
Sagittal plane data, degree							
Thoracic kyphosis	15.7 (8.1)	25.2 (5.8)	25.5 (7.0)	<0.001	<0.001	<0.001	1.000
Lumbar lordosis	47.0 (9.7)	45.0 (9.5)	50.2 (10.7)	<0.001	0.324	0.029	<0.001
Balance parameters and translational data, mm							
C7 translation from central sacral vertical line	16.9 (11.6)	14.8 (10.6)	9.5 (8.0)	0.005	0.798	<0.001	0.017
Sagittal vertical axis	−18.2 (18.8)	−7.7 (22.1)	−15.7 (21.3)	0.018	0.021	1.000	0.113
Thoracic apical vertebral translation	47.5 (16.8)	11.1 (9.4)	12.2 (8.3)	<0.001	< 0.001	<0.001	1.000
Thoracolumbar/Lumbar apical vertebral translation	16.3 (17.4)	11.8 (10.0)	11.2 (9.5)	0.779	-	-	-

The values are given as the mean (standard deviation).

## Data Availability

The data that support the findings of this study are available from the corresponding author on reasonable request.
